# A Comparative Study and Prediction of the Ex Vivo Permeation of Six Vaginally Administered Drugs across Five Artificial Membranes and Vaginal Tissue

**DOI:** 10.3390/molecules29102334

**Published:** 2024-05-16

**Authors:** Eleni Tsanaktsidou, Aikaterini-Theodora Chatzitaki, Anatoli Chatzichristou, Dimitrios G. Fatouros, Catherine K. Markopoulou

**Affiliations:** 1 Laboratory of Pharmaceutical Analysis, Department of Pharmacy, Aristotle University of Thessaloniki, 54124 Thessaloniki, Greece; etsanaktsi@pharm.auth.gr (E.T.); chatzica@pharm.auth.gr (A.C.); 2 Laboratory of Pharmaceutical Technology, Department of Pharmacy, Aristotle University of Thessaloniki, 54124 Thessaloniki, Greece; chatzita@pharm.auth.gr (A.-T.C.); dfatouro@pharm.auth.gr (D.G.F.); 3 Center for Interdisciplinary Research and Innovation (CIRI-AUTH), Aristotle University of Thessaloniki, 54124 Thessaloniki, Greece

**Keywords:** intravaginal permeability, in vitro/ex vivo absorption, PLS, Franz diffusion cells

## Abstract

The theoretical interpretation of the vaginal permeability phenomenon, the evaluation of the suitability of five artificial membranes, and the prediction of the behaviors of vaginal drugs were the main objectives of this study. Franz vertical diffusion cells and different validated HPLC methods were used to measure the permeability of six vaginally administered drugs (econazole, miconazole, metronidazole, clindamycin, lidocaine, and nonoxynol-9). This study was performed (in vitro) on different membranes of polyvinylidene fluoride (PVDF), plain cellulose or cellulose impregnated with isopropyl myristate (IPM), and cellulose combined with PVDF or IPM. The results were compared with those obtained from cow vaginal tissue (ex vivo), where cellulose was proven to be the best simulant. According to the permeability profiles (P_app_), the water solubility of the drugs was considered a necessary criterion for their transport in the membranes or in the tissue, while the size was important for their penetration. Furthermore, it was found that polar compounds show clear superiority when penetrating cellulose or tissue, while non-polar ones show superiority when penetrating the lipophilic PVDF membrane. Finally, a successful attempt was made to predict the P_app_ values (|P_app_-predP_app_| < 0.005) of the six drugs under study based on a PLS (Partial Least Squares) in silico simulation model.

## 1. Introduction

Until the beginning of the 20th century, the vaginal route of administration was considered unsuitable for the systemic absorption of drugs [1]. This perception was overturned by later studies where intravaginal administration was shown to be effective in systemic therapy as well [2].

Although this particular route of administration is characterized by gender limitation [3], its advantages (avoidance of side effects) allow for prolonged use (controlled release systems) with lower daily doses [1].

Anatomical characteristics that lead to the achievement of comparable pharmacodynamic results with oral administration are the large absorption surface of the vaginal tissue, the rich blood supply, the significantly reduced expression of phase I and II metabolic enzymes, and the sufficient permeability of drugs with various molecular weights [3,4,5]. 

Drug release, its dissolution in the vaginal fluid, and its permeability through the mucosa are the three steps involved in intravaginal administration. To optimize or prevent the above procedures, in vitro and ex vivo studies were developed using either artificial membranes or fresh/frozen human [6] and animal [7] tissues. In vivo studies are rare, as animal models involve additional expenditures in both time and cost as well as bioethical constraints [8]. There is no agreement in the scientific community on which animal model should be accepted for in situ/in vivo experiments. Therefore, several animals such as rats, guinea pigs, rabbits, monkey rhesus, cows, and sheep have been proposed [8,9]. In general, bovine vaginal mucosa was chosen as a suitable surrogate due to anatomical, physiological, and functional similarities to humans [10]. 

The use of Franz diffusion cells provides key insights into the assessment of permeability by defining the relationships between tissue, pharmaceutical active substance, and formulation [11]. Franz cells are usually employed in human or animal tissue for ex vivo experiments. However, when biological tissues are not readily available, synthetic membranes are recommended by the Food and Drug Administration (FDA) [12]. These are widely utilized by the scientific community to study the rate of permeation and/or release of an API from its formulation [13,14]. A wide variety of artificial membranes are commercially available, whose composition can be semi-synthetic to synthetic polymers. According to the literature, synthetic membranes of different materials, pore sizes, and thicknesses have been applied in various studies [15]. In general, for an in vitro evaluation of a potential drug or formulation, the selected membrane should allow the active ingredient to readily diffuse into the receptor as it is “released” from the dosage form. It should also provide an inert holding surface for the study formulation, but not act as a barrier [16]. Several factors should be considered when selecting a porous synthetic membrane for Franz cell studies, such as porosity, tortuosity, and thickness. If a membrane contains filler support, caution is needed to ensure that it does not interfere with drug flux and is compatible with the donor and acceptor components. Finally, the cost-effectiveness of the membranes is also considered important [17].

The diffusion of a drug depends on its molecular size, its interaction with the membrane, as well as the structural characteristics of both the molecule and the membrane [18]. 

Over decades, several studies have been published using Franz diffusion cells, aiming to determine the artificial membrane that better mimics the permeability phenomenon. A significant study focused on the transmembrane diffusion and release of ibuprofen through thirteen artificial membranes [17]. In a similar perspective, five different types of artificial membranes (polysulfone, cellulose, oxycellulose, nylon, Teflon, and polyanthracene) were examined in order to evaluate the permeability of semi-solid retinoic acid products (creams and ointments) [16]. 

Furthermore, the permeability of rivastigmine was assessed using synthetic membranes and tissue from pig ear (in vivo–in vitro correlation) [19]. In the same context, P. Clément et al. investigated the release of caffeine from w/o emulsions using three different synthetic membranes (silicone, cellulose, and polysulfone) [20]. Finally, in a similar study, a comparison was made between hydrophilic and hydrophobic synthetic membranes with human skin to evaluate the in vitro permeability of niacinamide [21].

It is noteworthy that there is no report in the existing literature comparing in vitro results with ex vivo results of intravaginal drug administration. Therefore, in an attempt to investigate the mechanism of the vaginal permeation of drugs, a series of experiments were performed on cow vaginal tissue adapted to Franz vertical diffusion cells. The same procedure was repeated on five artificial membranes (PVDF, cellulose, IPM-impregnated cellulose, and two hybrids of cellulose and PVDF or IPM-cellulose) in order to reveal which one can better simulate the tissue. The tested membranes were chosen based on their wide applicability in in vitro experiments, their lipophilic/hydrophilic characters, their wide commercial availability, and their low cost. When carrying out the method, six vaginal drugs were used (as probes) whose P_app_ values were determined both experimentally and computationally using the multivariate (Partial Least Squares) PLS model. Considering that the vaginal tissue consists of a non-keratinized squamous epithelium covered by approximately 0.50–0.75 g of fluid [22], an effort was made to interpret the phenomenon by determining the mechanism and differentiating it from the counterparts occurring in the skin or other tissues.

## 2. Results and Discussion

### 2.1. HPLC Methods

The chromatographic conditions of the analytical methods used for the separate quantification of each analyte and their linearity results are represented in Table 1.

**Figure 1:**
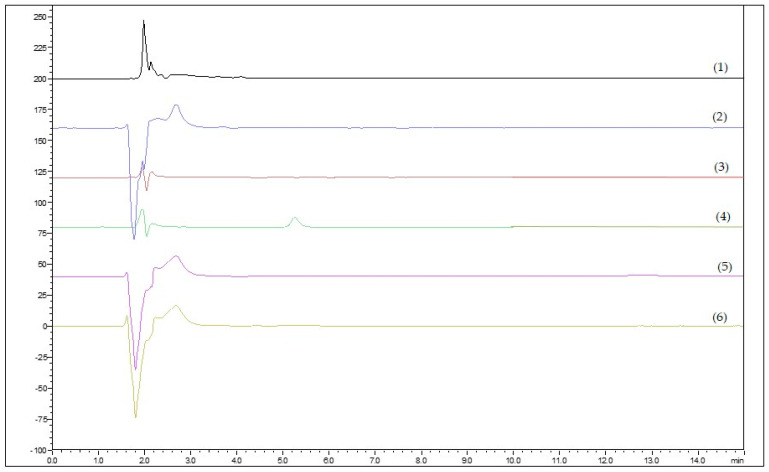
Chromatograms from blank on different membranes: (1) ex vivo, (2) PVDF-cellulose, (3) cellulose, (4) PVDF membrane, (5) IPM, and (6) IPM-cellulose. Retention time of METRO: 5.3 min; CLIND: 4.3; LIDO: 5.4 min; ECO: 4.5 min; MICO: 8.4 min; and NONO: 11 min.

In order to validate the methods, a series of analytical tests were carried out regarding the selectivity (Figure 1), repeatability, accuracy, and determination of LOQ (Limit of Detection) and LOD (Limit of Quantification) values (Table 1 and Table 2).

### 2.2. Permeability Studies

Six active pharmaceutical ingredients (ECO-econazole, MICO-miconazole, CLIND-clindamycin, LIDO-lidocaine, and NONO-nonoxynol-9), five synthetic membranes (with hydrophobic or hydrophilic characteristics), and bovine vaginal tissue were used to investigate the passive diffusion of drugs during intravaginal administration. To ensure the validity of the in vivo experiments, quantitative verification of the drugs was performed before and after the completion of the procedure (Table 3). 

Drug diffusion through synthetic membranes appears to be significantly affected by both their physicochemical properties (Table S1) and membrane composition and characteristics (Table S2).

PVDF is a polymeric membrane composed of polyvinylidene fluoride (CH_2_CF_2_) and is used for in vitro drug permeation studies. Hydrophobic interactions between its surface and drugs can affect both the rate and extent of their penetration, especially for molecules with lipophilic properties. 

As for the cellulose membrane, which is often used for in vitro experiments, although it is generally characterized as hydrophilic, its surface exhibits amphiphilic characteristics (consisting of polar –OH and nonpolar –CH groups). Its structural similarity to biological membranes (phospholipid bilayers) confirms its reliability as a marker in in vitro drug studies. The impregnation of cellulose membrane with isopropyl myristate (IPM) is a different approach that is often used in drug permeation studies [22]. IPM is a hydrophobic modifying agent that imparts lipophilic characteristics to the cellulose membrane, aiming to create conditions that may more closely resemble lipid-rich regions. Finally, to find the optimal simulation of vaginal tissue, two hybrid membranes are used, where cellulose (hydrophilic) is combined with either a PVDF (hydrophobic) or with an IPM-impregnated cellulose membrane. 

**Figure 2:**
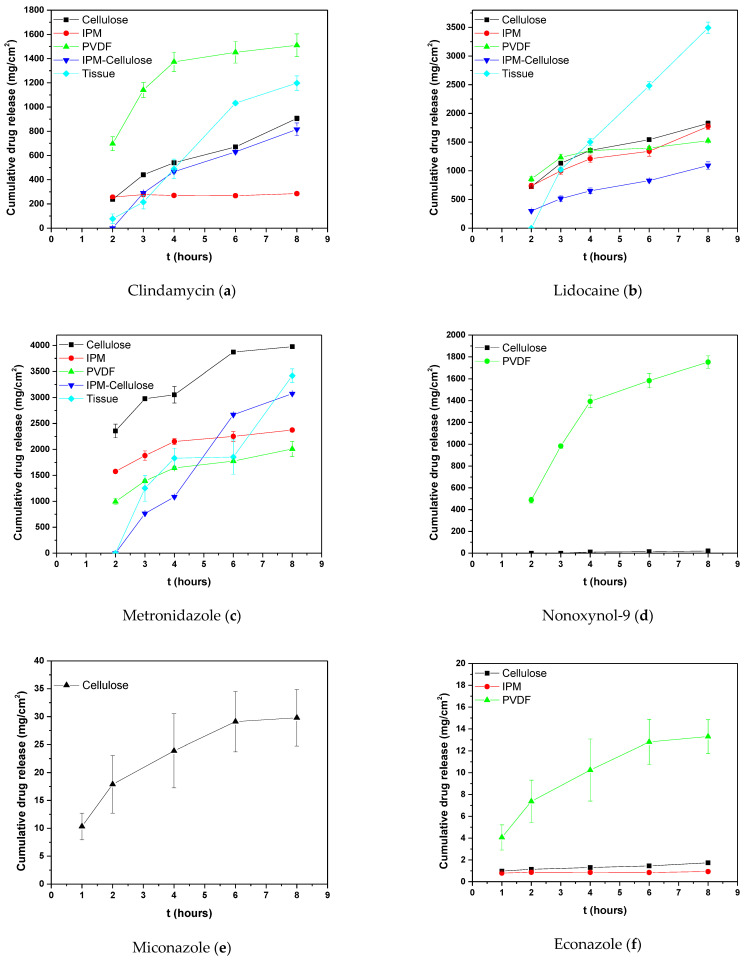
Cumulative amount released (Q) from each compound per unit area over time sampling. (**a**) Clindamycin, (**b**) lidocaine, (**c**) metronidazole, (**d**) nonoxynol-9, (**e**) miconazole, and (**f**) econazole.

The profile of the cumulative amount (Q) of each compound released per unit area at the sampling times is depicted in Figure 2, while the total amount of each substance that permeated the membranes after 8 h and the corresponding P_app_ values are listed in Table 4a and Table 4b, respectively. According to the experimental results, the hydrophilic cellulose membrane was considered the most suitable (reference membrane), as each compound showed a similar behavior (P_app_ and Q values) to the vaginal tissue. The cellulose membrane impregnated with lipophilic material (IPM) showed similar permeation behavior to the substances, but with relatively lower P_app_ and Q values. The PVDF showed a completely different permeability range for the compounds, with nonoxynol-9 being the compound with the second highest permeability (which does not penetrate the vaginal tissue at all), while the two hybrid membranes allow the substances to penetrate at lower concentrations and with a different priority order.

The Q plots of hydrophilic or relatively hydrophilic (logP < 2.5) compounds (clindamycin, lidocaine, and metronidazole) presented similar profiles with increasing permeation rates (mg/cm^2^) in all cases (artificial membrane or tissue), while the relatively lipophilic ones (nonoxynol-9, miconazole, and econazole) did not behave in the same way (Figure 2).

In a similar study conducted on 92 drugs (68 of 92 had logP < 2.5), the hydrophilic artificial membrane was found to provide a more reliable mimic of tissue penetration at levels comparable to Caco-2, and it was significantly superior to in silico techniques [23]. Also, such membranes require an overall shorter experimental time (in 2 h) compared to hydrophobic ones (10 h).

Accordingly, 10 different artificial membranes were used in Franz diffusion cells for the in vitro absorption study of nitroglycerin (log P = 1.6), where it was found that hydrophobic membranes impeded the diffusion of nitroglycerin into the receptor chamber and are therefore not recommended for use [24]. 

As seen in Table 4, the two substances (metronidazole and lidocaine) that first passed (highest permeability) through the animal tissue showed similar behavior on both artificial cellulose membranes and cellulose impregnated with IPM. The lipophilic membrane and the two hybrids (with amphiphilic character) did not provide similar results. Clindamycin is the third substance in the series, and it has a reduced ability to penetrate the vaginal tissue, although in general, its difference in physicochemical properties compared to the other two is not particularly great (Table S1). This can be interpreted based on Lipinski’s rule of five [25], according to which a compound should not have a molecular weight greater than 500 Da in order to penetrate a tissue. Obviously, because the molecular weight of clindamycin (M.W. 425) approaches the acceptance limit, permeation difficulties are encountered. Likewise, econazole (MW 479.1) and miconazole (MW 444.7) (with similar molecular weights) have zero permeation, while lidocaine (MW 234.34) and metronidazole (MW 171.15), with low molecular weights (Table 4 and Table S1), have satisfactory permeability. On the other hand, the diffusion profiles of the more hydrophobic compounds such as econazole, miconazole, and nonoxynol-9 were zero in both the vaginal tissue and cellulose-IPM membrane, while it was negligible in the cellulose membrane. The only membrane in which one of the substances (nonoxynol-9) showed numerically significant Q and P_app_ values was the hydrophobic PVDF.

In an extensive comparative study by Ng et al. [17], in five cellulose-based membranes and eight polymers, the transmembrane diffusion of ibuprofen (MW 206.28, logP 3.97) was investigated using Franz cells. The observations showed that cellulose-based membranes generally yielded lower flux, while polymer membranes showed a greater diffusion of ibuprofen due to their high lipophilicity. Overall, there were no statistically significant differences in ibuprofen flux (six hours) when membranes with different pore sizes or surface functional groups were used, except that some synthetic membranes acted more restrictively. It is therefore obvious that lipophilic substances diffuse more easily into lipophilic tissues and vice versa as long as all other penetration-related factors are satisfied.

The reasonable question that arises in this case is why the two lipophilic substances, econazole (logP 4.24, logS −4.34) and miconazole (logP 4.85, logS −5.81), despite their relatively low molecular weights (compared to nonoxynol-9, MW 616.8), do not diffuse through the PVDF membrane in the same way as nonoxynol-9. Permeability is a multiparametric phenomenon, and both the membranes/tissues and the physicochemical properties of the substances under study are equally important factors that will determine this phenomenon. In this case, the effect-determining property is their low solubility in water compared to that of nonoxynol-9.

According an our previous study [26], the most significant physicochemical properties that affect drug permeability are the polar surface area (PSA), the logP and logD values at pH 4.5 (negative impact), the water solubility logS (positive impact), flexibility (positive impact), and the molecular volume or weight (negative impact). From the values of the corresponding physicochemical properties listed in Table S1, it is evident that econazole (Molecular Flexibility 0.4146) and miconazole (Molecular Flexibility 0.4179) are the substances with the lowest water solubility and flexibility. The solubility of drugs in aqueous solutions, due to the hydrophilic nature of vaginal fluid, is a fundamental condition for their permeability.

It is known that 0.50–0.75 g of fluid is present in the vaginal cavity of a healthy woman of reproductive age (daily production: 6 g) [22]. Obviously, a key requirement for drug bioadhesion is its ability to reach the target through the aqueous carrier. Therefore, its poor solubility can usually be associated with limited bioavailability [27]. Of course, it should be noted that, in some cases, it is desirable that a drug should not penetrate the vaginal epithelium but remain on its surface. Indeed, compounds like econazole (logS −4.34) and miconazole (logS −5.81), whose solubility (logS) in aqueous solutions is 10 times lower than that of other drugs (Table S1), exhibited zero tissue permeability in the ex vivo experiments. More specifically, miconazole showed zero permeability, even in cellulose membranes, and econazole exhibited a P_app_ value of 0.00086, which is ten times lower than the permeability of other drugs. This happened because compounds with low solubility exhibit low transport capacity to the target tissue; thus, there is a lack of penetration into the tissue. Overall, there appears to be a solubility threshold that determines whether a drug candidate can enter general circulation via the vagina. At the same time, a property that probably contributes to the difficulty of the two substances reaching the tissue is their reduced flexibility. 

Among the substances studied, nonoxynol-9 (Table 4 and Table S1) has special behavior and properties since it is the most lipophilic, it has the largest volume and molecular weight, and it has great flexibility and solubility in water (Table S1). Due to its good miscibility with water and its flexibility, nonoxynol-9 certainly has the ability to approach the tissue/membrane, but due to its lipophilicity, it can only penetrate lipophilic surfaces (PTFE membranes). In a similar study [28], the permeability of the ingredients (niacinomide, ascorbic acid 2-glucoside, retinol, and polyethylene glycol-retinamide) was evaluated using a series of synthetic membranes and the Franz diffusion cell setup. Twelve different synthetic membranes were employed, revealing that niacinamide and 2-glucoside ascorbic acid exhibited a similar diffusion pattern, while retinol and polyethylene glycol-retinamide showed a different one. This observation may be attributed to their hydrophilic properties and aligns with the present research, highlighting the increased importance of lipophilicity.

We could say that size may not be more important than solubility, but it plays a decisive role (as evidenced by the phenomenon under study) since if the compound is too large, sufficient solubility does not ensure tissue penetration. However, when two compounds show similar logP values, then their molecular weights (MWs) will determine their tissue permeability. Thus, it could generally be said that a combination of logS > −4 and MW up to about 500 leads to a compound reaching its permeability tolerance limit.

In conclusion, the process of diffusion of a drug from the vaginal tissue into general blood circulation is described by three stages of approach transport and permeation. Properties related to the solubility, lipophilicity, flexibility, and molecular size of a drug are decisive, but at each stage, one class of properties has the primary role.

### 2.3. Penetration Prediction with In Silico Models

As part of the documentation of the present study, an attempt was made to predict the behaviors of the six vaginal drugs by applying two previous computational techniques proposed in the literature. The first involves the use of a reversed-phase C4 column and recording the retention times (tr) of the six drugs of interest [29], and the second refers to the application of a multivariate PLS software (version 9.0) [26].

According to an earlier study, the elution order of substances on a C_4_- RP-HPLC column is proportional to their penetration into a tissue [29]. Under the given conditions, it was assumed that the stationary phase simulates the tissue while the aqueous mobile phase represents the surrounding space. Indeed, when applying a corresponding experiment, the ascending elution order of the compounds was metronidazole, lidocaine, clindamycin, econazole, miconazole, and nonyxynol-9, which partly corresponds to their permeation order in the ex vivo experiments, as shown in Table 5. However, it should be clarified that under the present conditions, it was not possible to conduct a global assessment of the phenomenon because not all of the studied substances penetrated the tissue. However, this procedure could be used as an initial indication for the behaviors of potential drug candidates.

The in silico technique of partial least projections (PLS) is more powerful and has clearly superior predictive ability, and it was applied next. The data set of the model was mainly based on permeability information (Papp) derived from the experimental results of a previous study [26]. The method was applied to Franz cells on a cellulose membrane, which was considered to be the most suitable simulation of the atrial tissue.

The P_app_ values of the six vaginal drugs were determined using the model (Table 6), and their values were found to be in good agreement with the experimental ones.

## 3. Materials

The organic solvents (acetonitrile (ACN), and methanol) of the mobile phase were of analytical grade and purchased from VWR Chemicals (Radnor, PA, USA). The water had high purity (18.2 MΩ cm resistivity) and was produced using a B30 water purification system (Adrona SIA, Riga, Latvia).

For the preparation of the phosphate-buffered saline (PBS) with pH 7.4, in 1 L of distilled water, 0.24 g of KH_2_PO_4_ (Merck, Darmstadt, Germany), 0.20 g of KCl (Chem-Lab nv, Zedelgem, Belgium), 8.0 g of NaCl, 1.44 g of Na_2_HPO_4_, and 0.24 g of KH_2_PO_4_ (Merck, Darmstadt, Germany) were dissolved. Respectively, for the vaginal fluid simulant (VFS) with pH 4.5, they 0.222 g of Ca(OH)_2_ (Merck, Darmstadt, Germany), 3.51 g of NaCl, 1.4 g of KOH, 0.018 g of bovine serum albumin, 1.00 g of CH_3_COOH (acetic acid), 0.16 g of glycerol (Sigma-Aldrich, Darmstadt, Germany), 5.00 g of glucose, 0.40 g of urea (Fargon, Rotterdam, The Netherlands), and 2.00 g of C_3_H_6_O_3_ (lactic acid) were dissolved in 1 L of distilled water [22].

Polysorbate 80 (Tween 80) provided by Manis Chemicals (Athens, Greece) and polyethylene glycol 200 (PEG 200) obtained from Sigma-Aldrich (Darmstadt, Germany) were used as solubility enhancers.

**Figure 3:**
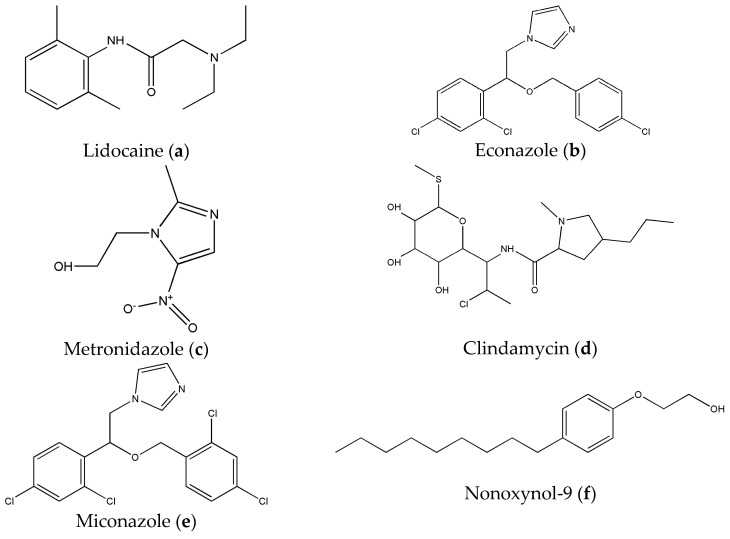
Chemical structures of studied compounds.

For the in vitro permeability experiments, cellulose membranes (obtained from Sig-ma-Aldrich (Darmstadt, Germany)) and PVDF (purchased from Labfill, Ljubljana, Slove-nija) were tested. Their technical characteristics are presented in the supplementary file (Table S2). Isopropyl myristate (IPM) was acquired from Sigma-Aldrich (Darmstadt, Germany). All compounds studied were United Stated Pharmacopeia (USP)-grade and obtained from Sigma-Aldrich (Darmstadt, Germany); their structures are represented in Figure 3. Cow’s vaginal mucosa was sourced from a local slaughterhouse. 

## 4. Methods

### 4.1. HPLC Analysis/Chromatographic Conditions

Chromatographic analyses were performed using a Shimadzu (Kyoto, Japan) HPLC system consisting of two LC-20AD pumps, a SIL-20AC HT autosampler (set at 20 µL), a CTO-20AC column oven, and an SPD-M20A diode array detector. 

The stationary phase involved a C_18_ Supelco Discovery^®^ (Darmstadt, Germany) column (150 mm × 4.6 mm, 5 µm) kept at 30 °C. The mobile phases were isocratic (flow rate 1 mL/min) and consisted of binary mixtures of suitable organic solvents and either water or aqueous buffer solutions (Table 1). Prior to the analysis, the system was equilibrated for approximately 20 min. Every analytical method was validated “in-house” according to the ICH (International Conference Harmonization) guidelines [30]. 

For the permeability study of drugs on the C_4_ column, special chromatographic conditions were applied. The analysis was carried out under isocratic conditions with a flow rate of 0.5 mL/min and a mobile phase consisting of MeOH-H_2_O at 10:90 (*v*/*v*). The analysis was conducted on an ACE column C_4_ (150 × 4.6 mm) with 5 μm at 40 °C. All analytes were dissolved in methanol at a concentration equal to 10 μg/mL, while the injection volume was adjusted to 20 μL [29].

### 4.2. Validation of Quantification Method 

The chromatographic methods were validated by testing the parameters of specificity, linearity, precision, accuracy, robustness, limit of detection (LOD), and limit of quantification (LOQ) [30]. 

Specificity was assessed by comparing blank samples (receptor medium) from blind permeability tests with the standard solution of each compound (Figure 1). Linearity was evaluated by a linear regression analysis of three replicates obtained at six different concentration levels (calibration curve concentrations ranged from LOQ to 50 μg/mL). The LOQ and LOD (Table 1) values were calculated based on the following equations:

LOQ = SD × 10/S,(1)

LOD = SD × 3/S,(2)

where SD is the standard deviation of the intercept with the y-axis and S is the slope. 

The accuracy of the method was determined at three concertation levels (Table 2), and the precision (repeatability) was determined by calculating the relative standard deviation (RSD) of six samples diluted in a receptor medium [31]. The RSD% values were <2%, and accuracies ranged from 98.37% to 102.8%. 

### 4.3. Drug Solubility in Donor Medium

After conducting the ex vivo permeability experiments, it was noted that the studied compounds did not permeate the vaginal tissue due to their low concentration (0.1 mg/mL). Normally, to find detectable amounts of drugs in the receptor chamber, their concentrations in the donor chamber should be in the range of 1–10 mg/mL. In order to ensure their high concentrations in the receptor chamber and to improve their solubility, a mixture of PEG 200-Tween 80 and VFS pH 4.5 (40:0.2:59.8% *v*/*v*) was used [26]. Their selection was based on the facts that these surfactants are compatible with vaginal administration [32], they are contained in intravaginal formulations [33,34,35], and they are listed in the comprehensive collection of pharmaceutical formulations in the vaginal category [36]. However, because a limited number of vaginal drugs have a water solubility of 10 mg/mL, two compounds with lower but satisfactory solubility values (econazole and miconazole) were also included in this study.

The solubility values of the six drugs were determined as follows: 5 mg of each compound was accurately weighed, and an appropriate amount (mL) of mix diluent was added until the compound was fully dissolved. The process was carried out at room temperature (25 °C) under constant gentle stirring. Their final concentrations were determined by HPLC analysis according to the methodology described in Section 4.1. 

### 4.4. Synthetic Membranes Preparation 

There are many membranes available on the market that are grouped as cellulosic and polymeric [17]. Taking into account those that are more convenient and economical, two synthetic membranes and their combinations were used. Synthetic PVDF (polyvinylidene difluoride) and cellulose acetate (Table S2) membranes [37] were pre-treated according to the supplier’s instructions. Cellulose membranes were further treated to acquire their lipophilic properties. Thus, they were placed on a flat glass surface and completely coated with IPM (isopropyl myristate) solution. The cellulose membrane was allowed to incubate in the IPM solution for 30 min. After impregnation, the excess IPM solution was drained off, and the membrane was used for the in vitro experiments. To ensure that the procedure was carried out successfully, the cellulose membrane was weighed before and after pretreatment [38].

### 4.5. In Vitro Permeability Studies

The in vitro permeability profile of each compound was evaluated using the Franz diffusion cell setup (PermeGear, Hellertown, PA, USA). Five distinct artificial membranes were placed between the donor (upper) and receiver (lower) chambers of cells, with an effective diffusion area of 4.9 cm^2^ and a receptor volume of 20 mL; 1 mL of APIs solutions were placed in the donor compartment, which was sealed with Parafilm^®^ to prevent any loss of moisture. The acceptor contained degassed PBS at pH 7.4. The procedure was performed under agitation at 90 rpm and at a temperature of 37 °C ± 0.2 °C. 

At specified intervals (t = 2, 3, 4, 6, and 8 h), 0.5 mL of the samples was taken from the receptor compartment and replaced with an equal volume of freshly prewarmed receptor medium. The concentration of each API was quantified using the appropriate HPLC method (Section 4.1) without sample pretreatment. Blanks containing PBS were pretreated with the same procedure. Each sample was analyzed in triplicate, and its cumulative release over time was plotted (Figure 2). The steady-state flux (Jss) was determined by plotting the permeation of the API per unit area (μg/cm^2^) against time (h) and calculating the slope of the linear portion of the resulting line. Equation (1) was applied to calculate the apparent permeability coefficient (cm/h) (P_aap_).

P_app_ = J_ss_/C_d_ (cm/h), (3)

where C_d_ is the initial concentration of the drug in the donor compartment and J_ss_ is the steady state flux.

### 4.6. Vagina Tissue—Ex Vivo Permeability Studies

Ex vivo experiments were conducted on cow vaginal mucosa sourced from a local slaughterhouse. Tissues with an exposure area of 0.19 cm^2^ were placed in a Franz diffusion cell with the epithelial mucosa side facing the donor compartment. In the donor compartment, 200 μL of API’s solution in VFS (pH 4.5) was added (37 °C ± 0.2 °C), while the acceptor was filled with 5 mL of PBS at pH 7.4 (constant stirring at 100 rpm). The concentration of APIs was adjusted to 10 mg/mL for clindamycin, metronidazole, nonoxynol-9, and lidocaine, while for econazole and miconazole, it was 1 mg/mL (Table 3). 

Samples were centrifuged at 2000× *g* for 10 min, and supernatants were analyzed using HPLC as described in Section 4.1.

The procedure for extracting the drug from the tissue was as follows: after the experiment was completed, each tissue was carefully removed from the Franz cell and immersed in 5 mL of methanol. It was then sonicated for 30 min, followed by 10 min of vertexing, another 30 min in the sonicator, and 10 min of vertexing. Finally, the samples were centrifuged for 20 min at 5000× *g*, and the supernatant was collected for analysis. The results are listed in Table 3.

### 4.7. PLS Methodology

The PLS model was developed using the Soft Independent Modeling of Class Analogy SIMCA P 9.0 program (Umetrics, Uppsala, Sweden) [39]. The database of the new model consists of 105 observations (3 outliers), 44 (X) variables (out of a total of 74), and one Y, the P_app_. Physicochemical properties of the compounds were calculated using a series of different software such as DataWarrior (version 6.1.0) [40] and ACD/Labs [41], or they were obtained from free online databases such as Pubchem [42]. Validation of the PLS models was performed using Cross-Validation (CV) and internal validation techniques (Table S3, Figures S1 and S2) [43]. After the validation of the PLS model, the significance and the positive or negative influence of each X-variable was estimated according to the VIP (Variables importance to the projection) and the (w × c [1] vs. w × c [2]) plots (Figures S3 and S4, respectively). 

## 5. Conclusions

As part of the study of drug permeability in vaginal tissue, in vitro and ex vivo experiments were conducted with both artificial membranes and bovine vaginal tissue. According to the results, cellulose was found to be the most suitable membrane as it exhibited similar behavior to the tissue, while the hybrid membranes failed to describe the phenomenon.

Regarding the efficiency of permeability, properties related to water solubility (logS) ensure the transport of molecules to the membrane/tissue, properties correlated to lipophilicity (polar surface area and partition coefficients “logP” and “logD”) facilitate their approach, and those connected to the molecular size (molecular weight and volume) determines their penetration. Finally, in silico PLS models proved that they could be useful tools for predicting and simulating such mechanisms.

## Figures and Tables

**Table 1:** Chromatographic conditions and regression analysis results.

Comp.	Calibration Curve	LOD (μg/mL)	LOQ (μg/mL)	R²	Mobile Phase	Retention Factor (k’)
NONO	y = 28002x − 56015	1.04	3.14	0.9993	20 mM KH_2_PO_4_ buffer, pH 3:MeOH, (20:80 *v*/*v*)	8.285
CLIND	y = 4887.3x − 743.42	0.08	0.25	1	20 mM KH_2_PO_4_ buffer, pH 3:ACN, (85:15 *v*/*v*)	1.061
LIDO	y = 11490x − 333.93	0.15	0.45	1	20 mM KH_2_PO_4_ buffer, pH 3:MeOH, (35:65 *v*/*v*)	0.728
METRO	y = 14236x − 1443.2	0.23	0.68	0.9999	20 mM KH_2_PO_4_ buffer, pH 3:MeOH, (80:20 *v*/*v*)	2.041
ECO	y = 90527x − 19250	0.34	1.02	0.9991	20 mM KH_2_PO_4_ buffer, pH 3:MeOH, (50:50 *v*/*v*)	2.105
MICO	y = 42121x − 9117.2	0.25	0.77	0.9996	20 mM KH_2_PO_4_ buffer, pH 3:MeOH, (30:70 *v*/*v*)	2.231

**Table 2:** The validation results of the model ingredients using the chromatographic conditions.

Comp.	Precision			Intermediate Precision				Accuracy		
	Theoretical (μg/mL)	av. Experimental Conc	RSD% of 6 Preparations	av. EXPERIMENTAL Conc		RSD% of 6 Preparations				
				2nd Day	3rd Day	2nd Day	3rd Day			
NONO	20	20.02 ± 0.33	1.67	20.1 (±0.2)	19.8 (±0.1)	1.5	1.8	40 μg/mL	15.5 μg/mL	5 μg/mL
								39.54 ± 0.14 (RSD: 0.36)/R%: 98.8	15.4 ± 0.2 (RSD: 1.4)/R%: 102.8	5.09 ± 0.02 (RSD: 0.38)/R%: 101.9
CLIND	5	5.01 ± 0.04	0.76	5.1 (±0.1)	4.8 (±0.3)	1	0.8	10 μg/mL	2.5 μg/mL	0.5 μg/mL
								9.91 ± 0.07 (RSD: 0.71)/R%: 99.1	2.48 ± 0.06 (RSD: 2.3)/R%: 99.5	0.49 ± 0.01 (RSD: 2.7)/R%: 99.7
LIDO	8	7.85 ± 0.02	0.21	7.92 (±0.1)	8.1 (±0.3)	0.8	0.6	12.5 μg/mL	5 μg/mL	1 μg/mL
								12.37 ± 0.02 (RSD: 0.13)/R%: 99.0	4.93 ± 0.01 (RSD: 0.18)/R%: 98.7	1.0 ± 0.01 (RSD: 0.81)/R%: 100.3
METRO	8	8.08 ± 0.02	0.23	7.95 (±0.1)	7.85 (±0.2)	0.5	0.8	12 μg/mL	5 μg/mL	1 μg/mL
								12.04 ± 0.04 (RSD: 0.4)/R%: 100.3	4.99 ± 0.01 (RSD: 0.11)/R%: 99.9	1.02 ± 0.01 (RSD: 0.18)/R%: 101.9
ECO	13	13.4 ± 0.06	0.48	13.1 (±0.03)	12.8 (±0.1)	0.7	0.9	15 μg/mL	5 μg/mL	1 μg/mL
								14.94 ± 0.04 (RSD: 0.3)/R%: 100.3	4.97 ± 0.03 (RSD: 0.2)/R%: 99.7	1.05 ± 0.03 (RSD: 0.25)/R%: 101.7
MICO	5.5	5.46 ± 0.06	1.14	5.3 (±0.04)	5.1 (±0.1)	0.9	1.0	15 μg/mL	5 μg/mL	1 μg/mL
								15.04 ± 0.07 (RSD: 0.27)/R%: 99.3	4.99 ± 0.01 (RSD: 0.7)/R%: 101.1	1.02 ± 0.01 (RSD: 1.1)/R%: 98.9

**Table 3:** Initial and final concentrations measured from ex vivo experiments and tissue extraction results.

Compound	Initial Donor Concentration (mg/mL)	Initial Mass Added (mg)	Final Mass Donor (mg) ^1^	Acceptor’s Mass (mg)	Tissue Extraction (mg)
CLIND	9.94	1.988	1.4 (±0.1)	0.23 (±0.07)	0.3 (±0.01)
ECO	1.24	0.248	0.2 (±0.01)	0	0.01 (±0.001)
LIDO	9.94	1.988	0.9 (±0.1)	0.53 (±0.09)	0.5 (±0.09)
METRO	9.98	1.996	0.01 (±0.001)	0.65 (±0.08)	1.4 (±0.2)
MICO	1.3	0.26	0.26 (±0.02)	0	0
NONO	10.04	2.008	1.85 (±0.2)	0	0.15 (±0.05)

^1^ Values are reported as mean ± standard deviation (n = 3).

**Table 4:** The results of the studied compounds over an 8 h period for (a) the apparent permeability coefficient (P_app_) and (b) the cumulative diffusion.

**(a)**							
**P_app_ (h/cm²) ^1^**							
	**Membranes**	**Cellulose** **(Hydrophilic)**	**Impregnated IPM**	**PVDF** **(Hydrophobic)**	**IPM-** **Cellulose**	**PVDF-Cellulose**	**Tissue**
**Compounds**							
LIDO		0.011 (±0.003)	0.011 (±0.003)	0.005 (±0.002)	0.012 (±0.001)	0.012 (±0.0007)	0.042 (±0.01)
METRO		0.023 (±0.0017)	0.014 (±0.0007)	0.009 (±0.003)	0.002 (±0.0001)	0.004 (±0.0009)	0.046 (±0.016)
CLIND		0.009 (±0.001)	0.0003 (±0.0001)	0.005 (±0.0007)	0.012 (±0.001)	0.015 (±0.0002)	0.02 (±0.0016)
NONO		2 × 10^−4^ (±3 × 10^−5^)	0	0.009 (±4.5 × 10^−5^)	0	0	0
MICO		0	0	0.0029 (±2 × 10^−5^)	0	0	0
ECO		8.6 × 10^−4^ (±2 × 10^−5^)	1.4 × 10^−4^ (±3 × 10^−6^)	0.00015 (±9 × 10^−6^)	0	0	0
**(b)**							
**Cumulative Amount Permeated (μg/cm^2^)**							
	**Membranes**	**Cellulose** **(Hydrophilic)**	**Impregnated IPM**	**PVDF** **(Hydrophobic)**	**IPM-** **Cellulose**	**PVDF-Cellulose**	**Tissue**
**Compounds**							
LIDO		1825.7 ^2^ 2nd (±44.9)	1772.0 2nd (±72.8)	1524.8 3rd (±18)	1091.9 1st (±91.9)	975.8 2nd (±61.8)	2789.3 2nd (±99.4)
METRO		3976.4 1st (±8.9)	2373.1 1st (±298)	2008.4 1st (±147)	307.3 3rd (±40.7)	370.2 3rd (±67.1)	3419.5 1st (±29.6)
CLIND		905.9 3rd (±38.8)	285.0 3rd (±48)	1510.1 4th (±188.6)	815.5 2nd (±85.2)	1047.1 1st (±22)	1197.5 3rd (±159.5)
NONO		20.1 4th (±1.1)	-	1752.2 2nd (±56)	-	-	-
MICO		-	-	33.4 5th (±3.07)	-	-	-
ECO		1.7 5th (±0.1)	0.92 4th (±0.02)	15.3 6th (±1.6)	-	-	-

^1^ The values are reported as the mean ± standard deviation (n = 3). ^2^ The index indicates the permeation order of the substance.

**Table 5:** Retention times (tr) of compounds in column C4.

Compound	tr (min)
CLIND	6.10
ECO	9.94
LIDO	5.94
METRO	3.93
MICO	10.2
NONO	39.48

**Table 6:** Experiment alvalues of P_app_ from in vitro experiments and PLS-predicted P_app_ values.

Compound	P_app_ (h/cm²)	YPredPS[3] (P_app_ (h/cm²))
ECO	8.6 × 10^−4^	−5.6 × 10^−4^ ≡ 0
METRO	0.023	0.027
MICO	0	−0.005 ≡ 0
LIDO	0.011	0.016
CLIND	0.009	0.007
NONO	2 × 10^−4^	8 × 10^−4^

## Data Availability

The original contributions presented in the study are included in the article/supplementary material, further inquiries can be directed to the corresponding author/s.

## Supplementary Materials

The following supporting information can be downloaded at: https://www.mdpi.com/article/10.3390/molecules29102334/s1. References [40,41,42,44] are cited in the supplementary materials.
