# Supplementary material for: A Comparative Study and Prediction of the Ex Vivo Permeation of Six Vaginally Administered Drugs across Five Artificial Membranes and Vaginal Tissue
Source: Molecules. 2024 May 16;29(10):2334. doi: 10.3390/molecules29102334 (PMC11123929; doi:10.3390/molecules29102334)
Supplement: Supplementary file 1 [file molecules-29-02334-s001.zip › molecules-2988179-supplementary.pdf]

Supplementary

Comparative study and prediction for the permeation of six intravaginally administered drugs across five artificial membranes and vaginal tissue *ex vivo*

Eleni Tsanaktsidou [1], Aikaterini-Theodora Chatzitaki [2], Anatoli Chatzichristou [1], Dimitrios G. Fatouros [2] and Catherine K. Markopoulou [1,*]

[1] Laboratory of Pharmaceutical Analysis, Department of Pharmacy, Aristotle University of Thessaloniki, 54124 Thessaloniki, Greece; etsanaktsi@pharm.auth.gr (E.T); chatzica@pharm.auth.gr (A.C.); amarkopo@pharm.auth.gr (C.K.M)
[2] Laboratory of Pharmaceutical Technology, Department of Pharmacy, Aristotle University of Thessaloniki, 54124 Thessaloniki, Greece; dfatouro@pharm.auth.gr (D.G.F.); chatzita@pharm.auth.gr (A.T.C.)
* Correspondence: amarkopo@pharm.auth.gr (C.K.M); Tel.: +30 2310 997665

Table S1. Physicochemical properties of the studied compounds

Comp.	MW[1]	logS[2]	LogP[2]	PSA[3]	Volume[4]	Relative PSA[2]	Tangible aqueous solubility [5]	Molecular flexibility[2] (low<0.5<high)
NONO	616.8	Miscible (-3.18)	4.82	103.3	590.8±3.0	0.19	miscible with H_2O (2.14 *10^{-7} g/L)	0.6797
LIDO	234.34	-2.369	2.15	32.34	238.8±7.0	0.14	very soluble (5.93*10^{-1} g/L)	0.5646
METRO	171.15	-0.2	-1.05	83.87	117.9±7.0	0.47	very soluble (5.92 g/L)	0.5058
CLIND	425.0	-2.28	0.91	127.56	327.2 ± 5.0	0.31	freely soluble (3.1 10^{-3} g/L)	0.5104
MICO	444.7	-5.81	4.85	27	296.0±7.0	0.09	very slightly soluble (7.63*10^{-4} g/L)	0.4179
ECO	479.1	-4.34	4.24	27	286.7±7.0	0.10	very slightly soluble (1.48*10^{-4} g/L)	0.4146

[1] g/mol, Pubchem [1], [2] Datawarrior [2], [3] Å^2, Pubchem, [4] cm^3, ACD (Advanced Chemistry Development) / Labs [3], [5] European Pharmacopeia

Table S2. Properties of the synthetic membranes

Properties / Membranes	MWCO (kDa)	Nature	Pore size (nm)	Thickness (μm)	Source
Cellulosic	0.500	Hydrophilic/nanofiltration	2.5	10^4	Sigma aldrich
PVDF	> 100 kDa	Hydrophobic/ microfiltration	220	125	Durapore® Millipore
IPM-impregnated	-	Hydrophobic	-	-	"In house"
Cellulosic/IPM-cellulosic	-	Hybrid	-	-	"In house"
Cellulosic/PVDF	-	Hybrid	-	-	"In house"

2. Development and Validation of PLS model

Initially, PLS was applied using 105 observations and 74 descriptors. Variables with low importance (VIP values < 0.5) and observations that were cosnidered as outliers were excluded and a more compact dataset with 102 observations and the 44 most relevant descriptors (Table S3) was revaluated (3 components). The compounds of interest were considered as test set of the model and predicted.

Table S3. Statistical parameters of PLS model

Model	R^2Y [1]	Q^2(cum) [2]	Number of Components	Excluded Observations (as outliers)	RMSE [3]	RMSEP [4]
P_{app}	0.658	0.574	3	**3**	0.00552743	0.00382278

[1] $R^2 = \sum_{i=1}^{N}(\hat{y}_i - y_i)^2 / \sum_{i=1}^{N}(y_i - \overline{y_i})^2$ ($\overline{y}_i$ represents the means of the true P_{app} values in the predictor set),

[2] $Q^2 = \frac{1-\text{PRESS}}{\text{SumSquares}}$, [3] RMSE = $\sqrt{\frac{\Sigma(\hat{y}_i - y_i)^2}{N}}$, ($\hat{y}_i$ represents the estimated Papp value for the ith object and y_i the reference P_{app} value), [4] RMSEP = $\sqrt{\frac{\Sigma(\text{obs} - \text{pred})^2}{N}}$.

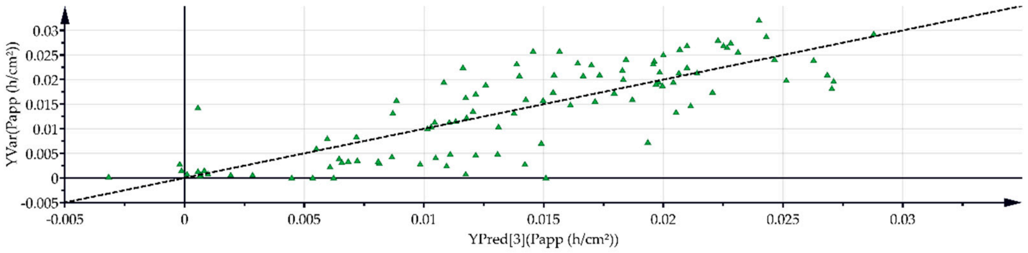

Figure S1. Observed versus estimated values of model P_{app} with *apparent permeability* values as (*Y*) variable.

During internal validation, the data for Y are not changed but they are randomly rearranged. Then the PLS model is applied again on the modified Y data and the R^2Y and Q^2Y values are recalculated. The above are compared with the initial values providing a first indication about the validity of the model. This process is repeated (20 permutations/model) and the resulting R^2Y and Q^2Y values are compared to the initial values to assess the validity of the model. In the diagram derived, the y axis represents the R^2Y/Q^2Y values of all models and the x axis represents the correlation coefficient between the modified and initial responses.

In order to summarize the results of the method, regression analysis is applied on both R^2Y and Q^2Y and the regression lines are obtained (Figure S2).

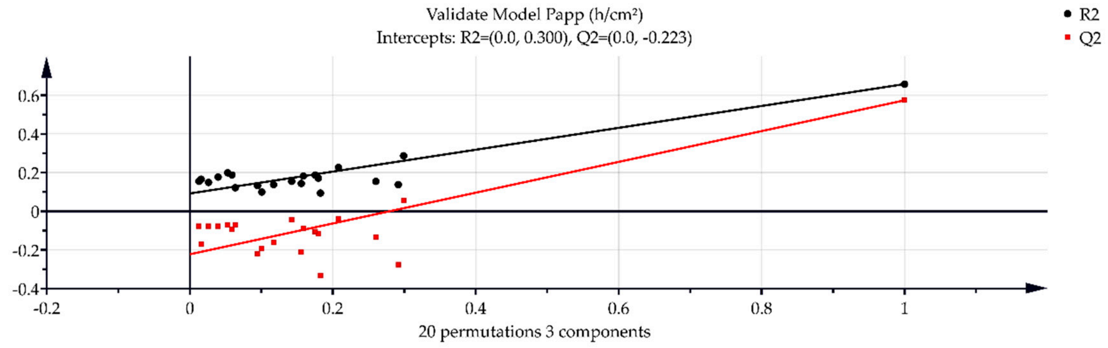

Figure S2. Internal validation test. Intercept limits: $R^2Y < 0.3\text{-}0.4$ and $Q^2Y < 0.05$ [4].

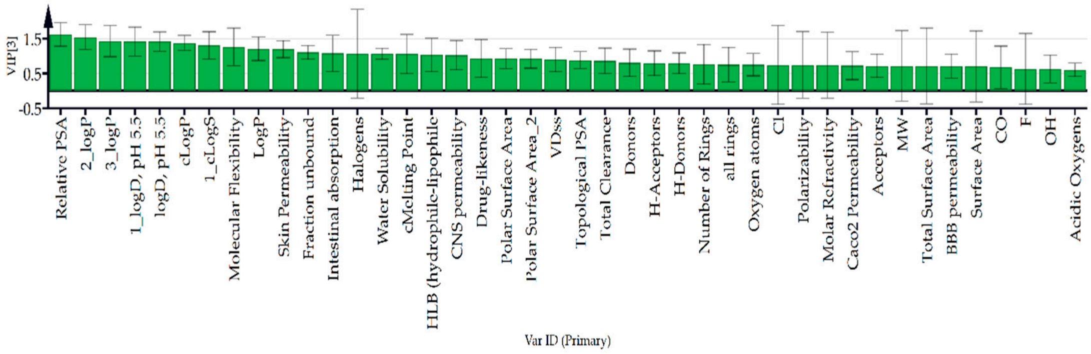

Figure S3. VIP values of selected descriptors in PLS models

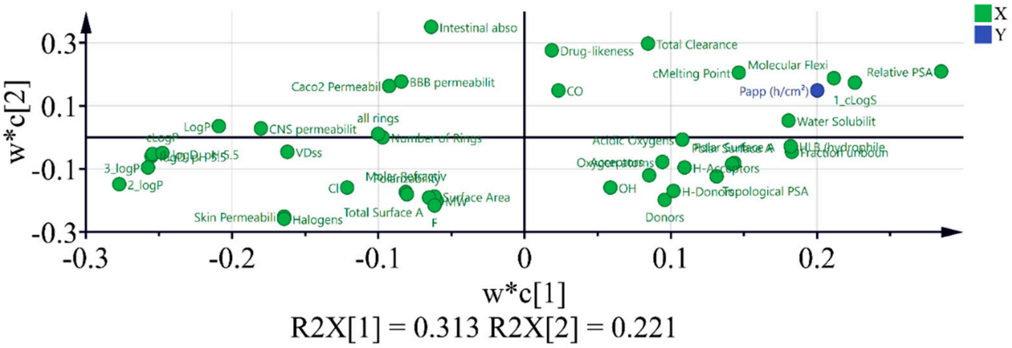

Figure S4. Scatter plot of w × c[1] versus w × c[2] for PLS model

References

[1] S. Kim *et al.*, "PubChem 2019 update: improved access to chemical data," *Nucleic Acids Res.*, vol. 47, no. D1, pp. D1102–D1109, Oct. 2018.

[2] T. Sander, J. Freyss, M. von Korff, and C. Rufener, "DataWarrior: An Open-Source Program For Chemistry Aware Data Visualization And Analysis," *J. Chem. Inf. Model.*, vol. 55, no. 2, pp. 460–473, Feb. 2015.

[3] "ACD/Labs," *Advanced Chemistry Development, Inc.*, 2015. [Online]. Available: https://www.acdlabs.com/index.php. [Accessed: 09-Sep-2019].

[4] D. M. Haaland, E. V. Thomas, D. M. Haaland, and E. V Thomas, "Partial least-squares methods for spectral analyses. 1. Relation to other quantitative calibration methods and the extraction of qualitative information," *Anal. Chem.*, vol. 60, no. 11, pp. 1193–1202, Jun. 1988.
